# The complete mitogenome of *Cylindrus obtusus *(Helicidae, Ariantinae) using Illumina next generation sequencing

**DOI:** 10.1186/1471-2164-13-114

**Published:** 2012-03-26

**Authors:** Dick SJ Groenenberg, Walter Pirovano, Edmund Gittenberger, Menno Schilthuizen

**Affiliations:** 1 Netherlands Centre for Biodiversity Naturalis, P.O. Box 9517, Leiden, RA 2300, The Netherlands; 2 BaseClear B.V., P.O. Box 1336, Leiden, BH 2302, The Netherlands; 3 Institute of Biology, Leiden University, Sylvius Lab., P.O. Box 9505, Leiden, RA NL 2300, The Netherlands

## Abstract

**Background:**

This study describes how the complete mitogenome of a terrestrial snail, *Cylindrus obtusus *(Draparnaud, 1805) was sequenced without PCRs from a collection specimen that had been in 70% ethanol for 8 years. The mitogenome was obtained with Illumina GAIIx shot gun sequencing. Although the used specimen was collected relatively recently and kept in a DNA-friendly preservative (not formalin as frequently used with old museum specimens), we believe that the exclusion of PCRs as facilitated by NGS (Next Generation Sequencing) removes a great obstacle in DNA sequencing of collection specimens. A brief comparison is made between our Illumina GAIIx approach and a similar study that made use of the Roche 454-FLX platform.

**Results:**

The mtDNA sequence of *C. obtusus *is 14,610 bases in length (about 0.5 kb larger than other stylommatophoran mitogenomes reported hitherto) and contains the 37 genes (13 protein coding genes, two rRNAs and 22 tRNAs) typical for metazoans. Except for a swap between the position of tRNA-Pro and tRNA-Ala, the gene arrangement of *C. obtusus *is identical to that reported for *Cepaea nemoralis*. The 'aberrant' rearrangement of tRNA-Thr and *COIII *compared to that of other Sigmurethra (and the majority of gastropods), is not unique for *C*. *nemoralis *(subfamily Helicinae), but is also shown to occur in *C. obtusus *(subfamily Ariantinae) and might be a synapomorphy for the family Helicidae.

**Conclusions:**

Natural history collections potentially harbor a wealth of information for the field of evolutionary genetics, but it can be difficult to amplify DNA from such specimens (due to DNA degradation for instance). Because NGS techniques do not rely on primer-directed amplification (PCR) and allow DNA to be fragmented (DNA gets sheared during library preparation), NGS could be a valuable tool for retrieving DNA sequence data from such specimens. A comparison between Illumina GAIIx and the Roche 454 platform suggests that the former might be more suited for *de novo *sequencing of mitogenomes.

## Background

Although NGS techniques advanced rapidly over the last years and sequencing of entire mitochondrial genomes (mitogenomes) has consequently become more common [1-3], knowledge about stylommatophoran ('terrestrial snails') mitogenomes seems to have advanced at a slower pace. For over two decades, the complete mitogenomes of only two stylommatophorans, *Cepaea nemoralis *[4] and *Albinaria caerulea *[5], had been known. Of a third species, *Euhadra herklotsi*, most of the mitogenome has been covered, so comparisons of mitochondrial gene arrangements could be made [6-8], but a complete sequence for that mitogenome is still missing [6,9,10]. Sequencing mitogenomes has been quite cumbersome because enrichment of the mitochondrial fraction (e.g. physical isolation of mitochondria, cloning of large mitochondrial fragments, long range, simplex or multiplex PCR) was quite laborious [1-3,8,11-15]. Moreover, the throughput of traditional (Sanger) sequencing is limited and sequencing of larger (> 1 kb) fragments is often delayed by the development of internal primers ('primer walking'). With NGS technologies, primer-directed amplification is no longer necessary and genome sequencing, mitogenomes in particular because of the small size and high-copy-number, has become fast and easy.

Nearly all traditional (Sanger) [1-3,8,11-15] and still some NGS [16] approaches for sequencing mitogenomes rely on long range PCR amplification. Due to the often degraded state of the DNA, this by and large excludes the use of specimens from natural history collections. An alternative is the hybridization capture approach [17], but this requires *a priori *sequence knowledge in order to design probes. Although enrichment of the mitochondrial fraction by long range PCR will increase the chance of obtaining a complete mitogenome (and facilitate the use of multiple specimens if sequence tags are exploited; [18]), it is not essential to NGS (e.g. [19-21]). In fact, the first step in NGS library preparation is fragmentation of the DNA. Consequently, DNA sequence data might be obtained from specimens of natural history collections with NGS, where PCR-based approaches fail. Whether NGS will allow, e.g. the recovery of complete mitogenomes from collection specimens, will depend on various parameters such as: the extent of DNA degradation, the ratio of nuclear to extrachromosomal DNA (which depends on the size of the genomes as well as on the type of tissue selected) and the number and length of the obtained sequences (dependent on the selected NGS platform). To the best of our knowledge, mitogenomes from NGS studies are thus far obtained either with the use of a long PCR enrichment procedure [16] prior to the NGS run, or with traditional Sanger sequencing after the run (to close the gaps remaining after assembly of the mitogenome, or to get an acceptable coverage) [19,20,22]. Since each of these approaches relies on PCR, both can be impracticable for (fragmented) DNA retrieved from museum specimens. With this study we wanted to test whether it would be feasible (using the Illumina GAIIx platform) to obtain a complete mitogenome, without PCR, from a single museum specimen.

**Figure 1:**
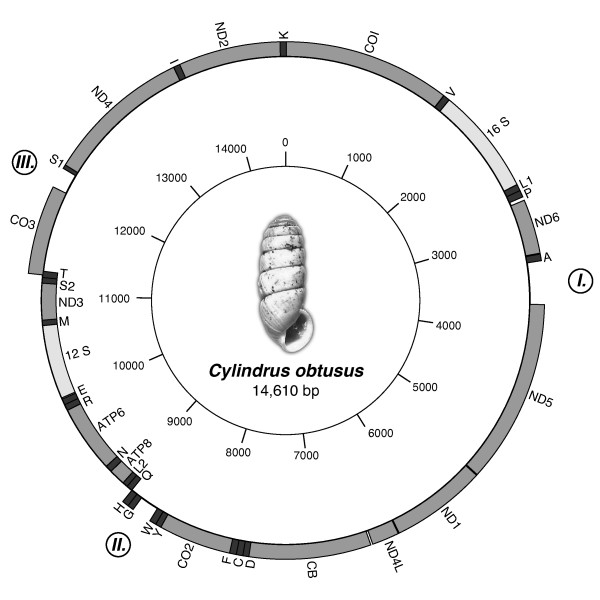
**Map of the mitochondrial genome of *Cylindrus obtusus *(GenBank accession nr. **JN107636). Genes on the outer circle are transcribed clockwise; genes on the inner circle are transcribed counterclockwise. TRNAs are denoted by their one-letter abbreviations. Regions I, II and III are regions that could not be assigned to any mitochondrial gene; Region III could be the most likely location for the mitochondrial control region (see Discussion).

We selected *C. obtusus *because it is an interesting species from both a morphological and a biogeographic point of view. *C. obtusus *is endemic to the Austrian Alps where it can be found in calcareous areas [23] at altitudes nearly always above 1,600 m. It has a disjunct distribution, probably mirroring the small insular areas ('nunataks'; [24]) in which it survived the last glacial maximum (LGM). Except for some Late Pleistocene specimens [25] there is no fossil record for *C. obtusus*. *Cylindrus *constitutes a monotypic genus. Within the helicid subfamily Ariantinae it is aberrant by being the only species with a cylindrical shell (Figure 1); other members of this speciose subfamily have broadly depressed (e.g. *Campylaea, Helicigona *and *Chilostoma*) or globular (*Arianta arbustorum*) shells (Figure 2 in [26]).

**Figure 2:**
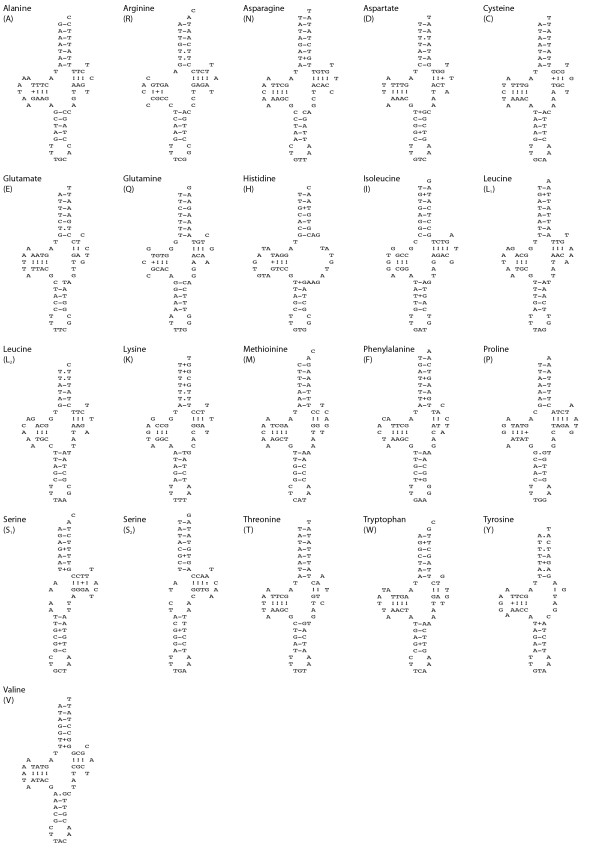
**Potential secondary structures of 21 inferred tRNAs of *Cylindrus obtusus *mtDNA**. Except for tRNA-Y, which was predicted by DOGMA, all tRNAs were predicted by ARWEN. The secondary structure for tRNA-G is missing, because it could not be predict by any of the programs tested.

This study shows that NGS can aid in the retrieval of sequence data (here a complete mitogenome) without using PCRs. Due to DNA degradation, PCRs are often a bottleneck for museum specimens. Based on our results for a specimen that has been in 70% ethanol for 8 years, we plea that NGS could be a promising technique for obtaining sequence data from museum specimens. We report the third complete mitogenome for a species of terrestrial snail ever published and compare our Illumina GAIIx strategy for sequencing mitogenomes with a similar study [20] in which the 454 platform of Roche was deployed.

## Methods

### Collection and preservation

Specimens of *C. obtusus *were collected by J. Gould in 2001 in Großer Buchstein (3.5 km NW of Gstatterboden), Ennstaler Alpen (Austria; 47°37' N 14°36' E) at an elevation of 2,200 m. After collection the specimens were drowned in water and subsequently placed in ethanol 70%. Finally they were stored in the molluscan wet collection of NCB Naturalis under collection number RMNH. MOL.144846.

### DNA extraction and quality assessment

DNA was extracted from a single specimen with a DNeasy Blood and Tissue kit (Qiagen). Apart from using a total of 40 μl of Proteinase K and overnight lysis, the manufacturer's instructions were followed. The DNA concentration of the extract was measured on a Nanodrop 1000 spectrophotometer (Thermo Scientific) and checked on an agarose gel.

### Confirmation of NGS output

Because only a small number of *C. obtusus *(microsatellite) sequences were present in GenBank [27], we tried to sequence *COI *and *CytB *which would allow the identification of mitochondrial contig sequences (expected GAIIx output). To do so, the following primers were selected: L1490 & H2198 for *COI *[28] and UCYTB151F & UCYTB270R for *CytB *[29]. PCRs were performed in 25 μl volumes using 1.5 mM MgCl_2_, 0.2 mM dNTPs, 0.4 mM of each primer and 0.25 μl (1.25 units) of Taq DNA polymerase (Qiagen). A thermoprofile of 3 min. at 94°C, followed by 40 cycles of 15 sec. at 94°C, 30 sec. at 50°C and 40 sec. at 72°C, and a final extension of 5 min. at 72°C was used for both markers. The PCR products were sent to Macrogen Europe (Amsterdam) where they were purified with a Montage purification kit (Millipore) and sequenced in both directions (using the same primers that were used for PCR) on an ABI3730XL. Contigs of forward and reverse sequences were assembled with Sequencher v. 4.10.1.

### Assessing limitations of the DNA extract: long range PCR

Although the aim of this study is to assess the possibility of sequencing a complete mitogenome without PCRs from a collection specimen, the underlying assumption is that enrichment of the target sequence(s) by primer-directed amplification will be difficult or impossible for these kinds of object. To test this assumption, we tried to enrich the mitochondrial fraction of the obtained DNA extract by means of long range PCR. To inrease the chance of successfully amplifying the complete mitochondrion, two *Cylindrus *specific primer sets (A and B) were designed that each amplified (roughly) half of the mitochondrion. These two primer-sets were tested with the "Expand Long Template PCR System" of Roche (Cat. No. 11 681 834 001), following the manufacturer's protocol. Primer-sets A and B were designed with Primer3 [30] and face outward of the obtained *COI *and *CytB *sequences:

A-Cobt-COI- 5'-TTACAACTATTTTTAATATGCGTTCTCCT-3' &

A-Cobt-CB- 5'-CGACGAGAAATAAAACATTTAACATAACTA-3' and

B-Cobt-CB- 5'- TACCTTTTGTGATTAGTGTTTTTGTGTTAT-3' &

B-Cobt-COI- 5'- TATTATTTATCCGGGGAAACCTTATATC-3'

We assumed that the orientation of *COI *and *CytB *would be identical to that of *C. nemoralis*.

### GAIIx library preparation

For DNA extracts from fresh tissues, the first step in library preparation is fragmentation of (genomic or PCR amplified) DNA. For extracts from museum specimens, DNA can already expected to be fragmented, which would make this step unnecessary or even detrimental ([17] and references therein). The extent of DNA degradation will depend heavily on the preservation history. Based on the quality assessment of our DNA extract and the adverse effect that improperly fragmented DNA has on GAIIx runs, we decided to follow a general library preparation procedure, for which we used the NEBNext™ DNA Sample Prep Kit (E6000-L, New England BioLabs). The DNA extract was randomly sheared with a nebulizer (K7025-05, Invitrogen) for 6 min. at 2.4 bar (35 psi) to obtain fragments in the range of 200-600 nucleotides. Fragments with a length of ca. 300 bp (insert-length without adaptors approx. 200 nucleotides) were extracted from an MS8-agarose gel (Talron Biotech. L.T.D.) with a Zymoclean Gel DNA Recovery Kit (Zymo Research, Orange, CA, USA). For all subsequent steps the NEBNext™ DNA Sample Prep Kit protocol was followed.

### GAIIx run and data analysis

The prepared library was run at BaseClear B.V. (The Netherlands) on a single lane of a GAIIx flow cell. CLC Genomics Workbench version 4.0 (CLC Bio, Cambridge, MA, USA) was used to filter the output and for *de novo *assembly.

### Annotation of the mitogenome

The contig sequence was annotated, based on similarity with available sequences in GenBank (Blast searches; http://www.ncbi.nlm.nih.gov:80/BLAST/), using pairwise alignments with, in particular, *C. nemoralis *and with the organellar genome annotation program DOGMA [31]. In order for DOGMA to detect all potential tRNAs (22 expected) we ultimately set the COVE-score to zero, but even then still some were missing. Therefore, the programs tRNAscan [32] and ARWEN [33] were used as well.

## Results

### DNA extraction

The DNA extract for the selected *C. obtusus *specimen had a yield 28 μg (200 μl buffer AE; concentration 139.8 ng/μl) and degradation as judged on the agarose gel was limited, considering the age of the specimen.

### PCR assessment

The PCRs for the 396 bp and 705 bp (including primer sequences) fragments of *CytB *and *COI*, respectively, worked on the first attempt and were directly sequenced. We failed to make the long PCRs work; with our *C. obtusus *specific primers (see Methods: Assessing limitations of the DNA extract), nor with any combination of the "universal" primers that successfully amplified the shorter *COI *and *CytB *sequences (L1490 & UCYTB270R; UCYTB151F & H2198).

### Illumina GAIIx run

The GAIIx run resulted in 34,174,164 reads with an average read length of 50 nucleotides. Of these 685,537 reads were overlapping and used for a *de novo *assembly (CLC Bio version 4.0). This resulted in 740 contigs with a total length of 478,878 bp. The largest contig had a length of 14,610 nucleotides and an average coverage of 26.65 ×. The latter contig was identified as the mitogenome of *C. obtusus *based on the expected length (roughly 14 kb), the presence of the *COI *and *CytB *sequences (see Methods: Confirmation of NGS output) and on similarity with mitochondrial sequences from other stylommatophorans as resulting from Blast searches.

### Initial assignment of PCGs and rRNAs

Twelve of the expected 13 protein-coding genes (PCGs), as well as both of the ribosomal RNAs (rRNAs) were recognized by DOGMA [31]; *ATP8 *had to be located based on a pairwise alignment with *C. nemoralis *and *A. caerulea*. Although the gene arrangement (of the PCGs and rRNAs) as assigned by DOGMA seemed correct (compared to the gene arrangement for *C. nemoralis*), the program had difficulties determining the gene boundaries (most likely due to the absence of similar sequences on GenBank). Since we lack data from peptide sequencing for any of the PCGs, the putative gene boundaries (Table 1) were determined based on pairwise alignments with the amino acid sequences of *C. nemoralis, A*. *caerulae *and *E. herklotsi*. Nine of the PCGs start with a common initiation codon (ATA 5×; ATG 4×); the other four start with less common (but not unique for invertebrates) initiation codons (TTG 2×; ATC 1× and GTG 1×). For four PCGs (unrelated to the four just mentioned) we had to infer that they ended with a truncated termination codon (that is, the stop codon is most likely generated by posttranscriptional polyadenylation; [5,34]).

**Table 1:** Summary of the mitochondrial genome content of *C.obtusus*

		Position							
								
Name	One letter code	From	To	Size	Strand	Nr. of amino acids	Anti- codon	Inferred initiation codon	Inferred termination codon
*COI*		1	1527	1527	H	508		TTG	TAA
*tRNA-Val*	V	1529	1589	61	H		TAC		
*16 S*		1590	2572	983	H				
*tRNA-Leu*	L1	2573	2635	63	H		TAG		
*tRNA-Pro*	P	2632	2694	63	H		TGG		
*ND6*		2727	3224	498	H	165		ATA	TAG
*tRNA-Ala*	A	3232	3293	62	H		TGC		
**Region *I***		3294	3688	395					
*ND5*		3689	5368	1680	H	559		TTG	TAG
*ND1*		5377	6249	873	H	290		ATA	TAG
*ND4L*		6258	6507	250	H	83		ATA	T
*CB*		6526	7651	1126	H	375		ATG	T
*tRNA-Asp*	D	7637	7696	60	H		GTC		
*tRNA-Cys*	C	7692	7752	61	H		GCA		
*tRNA-Phe*	F	7752	7814	63	H		GAA		
*CO2*		7814	8500	687	H	228		ATG	TAG
*tRNA-Tyr*	Y	8475	8528	54	H		GTA		
*tRNA-Trp*	W	8529	8590	62	H		TCA		
**Region *II***		8591	8771	181					
*tRNA-Gly*	G	8772	8832	61	H		TCC		
*tRNA-His*	H	8828	8889	62	H		GTG		
*tRNA-Gln*	Q	8891	8949	59	L		TTG		
*tRNA-Leu*	L2	8947	9004	58	L		TAA		
*ATP8*		8999	9157	159	L	52		GTG	TAG
*tRNA-Asn*	N	9158	9222	65	L		GTT		
*ATP6*		9222	9873	652	L	216		ATG	T
*tRNA-Arg*	R	9874	9935	62	L		TCG		
*tRNA-Glu*	E	9933	9992	60	L		TTC		
*12 S*		10004	10717	714	L				
*tRNA-Met*	M	10718	10779	62	L		CAT		
*ND3*		10770	11126	357	L	118		ATA	TAA
*tRNA-Ser*	S2	11123	11177	55	L		TGA		
*tRNA-Thr*	T	11177	11237	61	L		TGT		
*CO3*		11202	12014	813	H	270		ATG	TAA
**Region *III***		12015	12203	189					
*tRNA-Ser*	S1	12204	12257	54	H		GCT		
*ND4*		12240	13568	1329	H	442		ATC	TAA
*tRNA-Ile*	I	13569	13631	63	H		GAT		
*ND2*		13628	14606	979	H	326		ATA	T
*tRNA-Lys*	K	14559	7	59	H		TTT		

### Annotation of rRNAs

The conserved regions at the beginning and end of both rRNAs (described in Figure 4 in [5]) were found in the contig sequence of *C. obtusus *as well. However, for both *A. caerulea *and *C. nemoralis*, the annotation of rrnS (*12S*) and rrnL (*16S*) extends beyond these conserved regions. Even though the exact gene boundaries of these ribosomal genes need to be confirmed by transcript mapping, the sequence data for *A. caerulea *and *C. nemoralis *show little to no space between the rRNAs and the surrounding tRNAs. Consequently, we based the putative boundaries of *12S *and *16S *(Table 1) on alignments with sequences of the just mentioned species for those genes and the position of the surrounding tRNAs (described in the next paragraph).

### Annotation of tRNAs

Because of the low COVE-score, half of the tRNAs assigned by DOGMA (17 out of 34) were false positives, but even with these relaxed settings tRNA-P(Pro), -G(Gly), - S_2 _(Ser), - I(Ile) and -K(Lys) were missed. None of the missing tRNAs could be detected with tRNAscan. Using the least restrictive parameters, tRNAscan yielded no false positives, but merely detected four tRNAs (L_1_(Leu), -N(Asn), -M(Met) and -T(Thr)) that were already assigned by DOGMA. Of the three programs tested, only Arwen found 20 of the 22 tRNAs at the cost of just one false positive. When the output of ARWEN and DOGMA was combined, all tRNAs except tRNA-G(Gly) were assigned. Apart from a swap between tRNA-P and tRNA-A(Ala), the gene order for *C. obtusus *is identical to that of *C. nemoralis*. Alignments that included sequences of both *A. caerulea *and *C. nemoralis *[6,7] showed that tRNA-G is located between tRNA-W(Trp) and tRNA-H(His). In the annotated mitochondrion of *C*. *obtusus *(Figure 1), there is indeed an unassigned region between tRNA-W and tRNA-H. An alignment with sequences of *C. obtusus, A. caerulea *and *C. nemoralis *showed a conserved sequence, TACCTTCCAAG (8797-8809) within this non-annotated region of *C. obtusus*, which represents the anticodon loop and part of the anticodon stem of tRNA-G. Consequently, this alignment was used to infer the approximate location of tRNA-G, even though the secundary structure for this tRNA could not be predicted. Additionally, the location of each tRNext-link-id=""A was confirmed by the presence of the anti-codon. A map of the mitogenome of *C. obtusus *is depicted in Figure 1. A summary of the mitochondrial genome content is given in Table 1. The corresponding annotated sequence was deposited in Genbank under accession number JN107636.

## Discussion

At present, NGS techniques are being used increasingly in mitogenomic studies. One of the commonly used platforms is Roche 454 [16,19,20,22]. The ability to generate longer reads (which facilitates *de novo *assembly) and the reduction in sequence costs as compared to Illumina GAIIx, undoubtedly has added to the popularity of this platform. An overview of the costs and read lengths of these (and other) NGS platforms is given in Glen *et al. *[35]. When sequencing mitogenomes from museum specimens [21,36] the ability to sequence longer DNA fragments likely is of no advantage and such studies generally rely on platforms that are optimized for short fragments, such as Illumina GAIIx. Despite the momentum that NGS has provided for ancient DNA research [17], the number of mitogenomic studies that actually use these new techniques to exploit natural history collections still is rather limited. This paper provides an example of how NGS technology can be used to retrieve genetic information from a museum specimen. We show that it is feasible to sequence a complete mitogenome, without PCR, from a snail that has been in 70% ethanol for eight years.

Thus far, to the best of our knowledge, only [20] made use of an NGS platform to sequence the complete mitogenome of another gastropod. In that study, a similar approach (no prior enrichment of the mitochondrial fraction) was taken, albeit with freshly collected specimens and a different NGS platform (Roche 454-FLX). Despite the commonalites, there were some noteworthy differences between both studies as well. Firstly, [20] used 13 specimens to obtain a complete mitogenome, whereas our results were obtained from a single specimen. The rationale behind using this many specimens is not given by the latter authors; perhaps they wished to account for intraspecific heterogeneity between the selected populations. It had nothing to do with the 'sensitivity' of the different platforms; the amount of DNA that Feldmeyer *et al. *[20] used for their sample preparation (6 μg), was roughly similar to what was used in this study (5 μg). The ability to obtain a complete mitogenomic sequence from a single specimen obviously is an advantage. Secondly, in contrast to our GAIIx run, the 454 run did not cover the complete mitogenome, requiring the design of additional primers and Sanger sequencing to close the three gaps that were left over after assembly. This is also observed in other mitogenomic studies in which the 454 platform was used without prior enrichment of the mitochondrial fraction [19,22]. After filtering, the 454 run resulted in 114 reads with an average length of 318 nt [20] that could be assigned to the mitochondrial genome, compared to 7,808 reads (out of 34,174,164) with an average length of 50 nt obtained with GAIIx. Thus the 454 run resulted in 36,252 mitogenomic nucleotides, whereas our GAIIx run yielded 390,400. The maximum coverage obtained with 454 and GAIIx is 2.6× and 26.7× for *R. balthica *and *C. obtusus *for mitogenome sizes of 13,993 bp and 14,610 bp, respectively. Generally longer reads as obtained with the 454 platform facilitate *de novo *assembly and might be preferred when little *a priori *sequence information is available ([37] and references therein). When reconstructing the just mentioned mitogenomes the sheer number of short reads generated by the GAIIx platform outcompeted the smaller number of longer reads as obtained with 454 sequencing.

Identification of tRNAs in nematodes [38] and stylommatophorans [4-6] can be difficult because the standard cloverleaf secondary structure may not be present (T or D arms can be lacking; see tRNA-H, -S_1 _and -S_2 _in Figure 2) and pulmonate tRNAs can undergo post-transcriptional processing [7,39]. In a number of instances different tRNAs were predicted in the same approximate nucleotide region, depending on the algorithm used. The hypothetical tRNAs differed a few nucleotides in length, or were shifted a few bases, or both, causing a slight shift in the anti-codon region. Despite the fact that only one of the tRNAs will be real, both algorithms correctly point to (roughly) the same nucleotide region for the placement of a tRNA. In other instances, the different algorithms predicted tRNAs on exactly the same position but on opposite strands, causing the tRNAs to be the reverse-complement of each other [40]. Examples of the latter within *C. obtusus *are predictions by DOGMA of tRNA-W and tRNA-C on position 3632-3694 and 3232-3293, for which ARWEN predicted tRNA-P and tRNA-A, respectively. For both tRNAs, only those predicted by ARWEN were in agreement with existing annotations for related species in GenBank.

**Figure 3:**
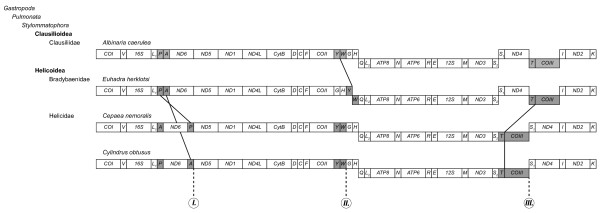
**Comparison of the gene order of the four known stylommatophoran mitogenomes**. For each mitogenome genes above the horizontal line are transcribed from left to right; genes below the horiziontal line are transcribed from right to left. TRNAs are denoted by their one-letter abbreviations. The diagonal lines indicate mitochondrial gene rearrangements between the specified taxa. In the gene-map of *C. obtusus, I-III *indicate three unassigned regions (see Figure 1 and Discussion). Gene sizes are not drawn to scale.

Although gene rearrangements are common in the mitogenomes of molluscs [7,8] and gastropods in particular [2,3,41], little is known about the mitochondrial gene organisation of terrestrial snails. The mt gene arrangements depicted by Yamazaki *et al. *[6] and Boore *et al. *[8] show less similarity between *C. nemoralis *and *E. herklotsi *(both species belonging to the Helicoidea), than between each of them and *A. caerulea *(a species belonging to the Clausilioidea). Based on a three-taxon statement Yamazaki *et al. *[6] concluded that the rearrangement of the tRNAs between *COII *and *ATP8 *represented a derived state in *E*. *herklotsi*. Similarly, they concluded that the positions of tRNA-P and the gene-region tRNA-T/*COIII *represented a derived state in *C. nemoralis*. By comparing these gene rearrangements with the gene order observed in *C. obtusus*, we can get some insight in the evolution of the mitochondrial gene order of the Helicoidea. Figure 3 gives an overview of the gene organisation of the four stylommatophoran mitogenomes currently known. Starting at *COI *the first observed rearrangement is the relocation of tRNA-P within *C. nemoralis*. Although the location of tRNA-P in *C. obtusus *is the same as that found within the other stylommatophorans, the gene region itself seems to be of high potential for rearrangements within the Helicidae. In *C. obtusus *we see that instead of tRNA-P, tRNA-A has been relocated between *ND6 *and *ND5*. Thus within the Stylommatophora, the relocation of a tRNA from the L_1_, A, P-region to between *ND6 *and *ND5 *seems to be a synapomorphy for the Helicidae. The second gene rearrangement (Figure 3) is found in *E. herklotsi *within a series of tRNAs located between *COII *and *ATP8*. Despite the fact that these tRNAs (Y, W, G, H, Q, L_2_) have rearranged frequently during gastropod evolution [2,3], the gene order seems rather conserved within the Eupulmonata. The five eupulmonates currently listed in the genome database of NCBI and *C. obtusus *all show the order Y, W, G, H, Q, L_2_. Therefore, we endorse the conclusion of Yamazaki *et al. *[6] that the arrangement of these tRNAs as observed in *E. herklotsi *can be considered a derived state (and possibly represents an apomorphy for the Bradybaenidae). Presence of the ancestral tRNA arrangement in both *C*. *obtusus *and *C. nemoralis*, suggests that that gene order has not changed in the Helicidae. The last gene rearrangement is the relocation of the gene-region tRNA-T/*COIII *in *C. nemoralis *from between *ND4 *and *ND2 *to between *ND3 *and *ND4*. Exactly the same rearrangement is also observed in *C. obtusus*, likely indicating an apomorphy for the Helicidae.

Most metazoan mitogenomes possess a single major non-coding region presumed to contain the signals for replication and transcription. This region is usually referred to as the control region [42]. In some groups of invertebrates, such as gastropods [2,5] and spiders [43] the mitogenomes can be very compact, hardly leaving any non-coding regions of significant length. Although the mitogenome of *C. obtusus *(14,610 bp) is still compact compared to that of other Metazoa (approx. 15-24 kb; [44]), it is about half a kb larger than the mitogenomes of *C. nemoralis *(14,100 bp) and *A. caerulea *(14,130 bp). The schematic overview of this mitogenome (Figure 1) shows three unassigned regions (indicated as *I, II *and *III*) with a length of 394, 181 and 189 nt, respectively. When the mitochondrial gene order of *C. obtusus *is compared with that of other stylommatophorans currently known (Figure 3), it becomes clear that region *I *and *III *coincide with the transposition of tRNA-A and the region tRNA-T/*COIII*. As for region *II*, no gene rearrangement was observed in *C. obtusus *and neither *C*. *nemoralis *nor *A. caerulea *have any unassigned sequence between tRNA-W and tRNA-G. But then *E. herklotsi *does show a gene rearrangement in that region (Figure 3). In stylommatophorans (or all gastropods for that matter) the location of the mitochondrial control region is still a subject of debate. Given the absence of region *II *in other stylommatophorans, we believe that of the three non-coding regions, region *II *is the least likely location for the control region. As for region *I*, Grande *et al. *[9] suggested that the region between *ND6 *and *ND5 *might contain recognition signals for transcription in the nudibranch *Roboastra europaea*. Except for *Onchidella celtica*, which, like *C. obtusus*, has its longest non-coding sequence here [2], most of the heterobranch gastropods sequenced thus far show very little unassigned sequence between *ND6 *and *ND5*. Therefore we assume that region *I *is not the most likely location for the control region either. Within the heterobranch gastropods the region between *COIII *and tRNA-I is most often cited as the potential location for the control region [2,3,9,45]. Because of the shown transposition of tRNA-T/*COIII *within *C. nemoralis *and *C. obtusus *(Figure 3), this region was transposed as well. Thus for those species (and likely all Helicidae), the potential control region (the region adjacent to *COIII*) is not located between *COIII *and tRNA-I, but between *COIII *and tRNA-S_1_. For *A. caerulea *and *Pupa strigosa*, the presence of 10 nt (or 20 nt if the 5 nt overlap with tRNA-I is included) and 25 nt palindromes, respectively [5,45], has been implicated to function as a bidrectional promotor for this putative control region. Within *C. obtusus, C. nemoralis *and *E. herklotsi*, the palindromes found in this region were never larger than six nucleotides. We were unable to confidently align the putative control region for the four mentioned stylommatophorans. The length of this region was longer in the Helicidae (*C. obtusus *= 189 nt; *C. nemoralis *= 158 nt) than in the other two stylommatophorans (*A. caerulea *= 42 nt; *E. herklotsi *= 43 nt), which is most certainly related to the transposition of tRNA-T/*COIII*. Besides the apparent consistency of the presence of a non-coding region adjacent to *COIII*, there still is little to go on for recognition of the control region in heterobranch gastropods. Given the limited number of available stylommatophoran (and even eupulmonate) mitogenomes (GenBank), we consider more extensive genomic comparisons (such as [46]) to be premature, based on these data.

Although this study illustrates the potential of NGS to obtain genetic information from museum specimens, there are some caveats that need to be addressed. As for the results of the long range PCR, we did not have a recently collected specimen of *C. obtusus *at our disposal. Therefore, we have not shown that the failure of the long range PCR was caused by the fact that we used an 'aged' collection specimen. Otherwise, no products would have been obtained with the long range PCRs either, based, as they were, on the universal primers, which were shown to work with *C. obtusus*. The fact that an NGS approach worked well in this study, also does not imply that NGS approaches will always be fruitful when applied to collection specimens. A vast number of parameters such as the effect of fixatives, time (the "age" of specimens) and preservation history were not assessed in this study. Also annotation of a mitogenome largely based on similarity with available sequences in GenBank (as opposed to transcript mapping or peptide sequencing) is hazardous. Closely related species might not be present and existing annotations are not guaranteed to be flawless [47,48]. Another hypothetical problem is that due to the relative short length of the obtained sequences (50 nucleotides on average) repeats within the mitogenome could be missed. The mitogenomes of gastropods however are very compact, none of stylommatophorans sequenced thus far show such repeats and the length of the complete mt sequence is similar to that of other stylommatophorans. We therefore assume that the mitogenome of *C. obtusus *presented here is complete. Based on the sheer number of sequences generated with GAIIx and 454, we are convinced that without PCRs it will be more difficult to obtain a complete mitogenome with the latter platform (despite the longer reads). Even though our GAIIx approach is reasonably similar to the 454 approach described by Feldmeyer *et al. *[20], the comparison will not be conclusive as long as the total genome sizes of *C. obtusus *and *R*. *balthica *are unknown. The average genome size (http://www.genomesize.com) of available stylommatophorans (2.86 c or 2.79 GB) compared to basommatophorans (1.34 c or 1.31 GB) nevertheless suggest that it will be more difficult to obtain the mitogenome of *C. obtusus *than that of *R. balthica*. It is likely that the assembly of mitogenomes will benefit from the advances in NGS technologies (e.g. the Illumina HiSeq platform), as well as from the promising arrival of third generation sequencers (e.g. the PacBio RS platform).

## Conclusions

On a par with previous studies [21,36], this study shows that NGS can aid in the retrieval of mitogenomes from museum specimens. Although sequencing of mitogenomes by means of NGS without an enrichment procedure is very inefficient (only 0.02% of the reads from our GAIIx run were used for assembly of the mitogenome), it eliminates the use of PCRs which is often a bottleneck for degraded DNA samples. Without prior enrichment of the mitochondrial fraction, the GAIIx platform (Illumina) might be better suited for *de novo *sequencing of mitogenomes than the 454 platform (Roche). Besides being much faster than conventional sequencing (which generally results in 2× coverage), sequencing of mitogenomes by means of NGS also yields higher confidence estimates (on average 26× times coverage, in this study). Except for a swap between tRNA-P and tRNA-A, the mitochondrial gene arrangement of *C. obtusus *is identical to that of *C. nemoralis*. Within the Helicidae the region tRNA-L_1_, P, A might be a hot spot for transposition of genes (in particular to the region between *ND6 *and *ND5*). The location of tRNA-T/*COIII *between *ND3 *and *ND4 *(instead of between *ND4 *and *ND2*) might be an apomorphy for the family Helicidae. We hope that the results of this study will aid to future studies on stylommatophoran evolution and the phylogeny of the subfamily Ariantinae in particular.

## Competing interests

The authors declare that they have no competing interests.

## Authors' contributions

DSJG conceived and designed the study, carried out the DNA extraction, performed the annotations and wrote the manuscript. WP carried out the mitogenome assembly and helped to draft the manuscript. EG and MS participated in the design of the study and helped to draft the manuscript. All authors read and approved the final manuscript.

## References

[B1] Saitoh K Sado T Mayden RL Hanzawa N Nakamura K Nishida M Miya M Mitogenomic evolution and interrelationships of the Cypriniformes (Actinopterygii: Ostariophysi): the first evidence toward resolution of higher-level relationships of the world's largest freshwater fish clade based on 59 whole mitogenome sequences J Mol Evol 2006 63 6 826 841 10.1007/s00239-005-0293-y 17086453

[B2] Grande C Templado J Zardoya R Evolution of gastropod mitochondrial genome arrangements BMC Evol Biol 2008 8 61 10.1186/1471-2148-8-61 18302768 PMC2291457

[B3] Lal S Vallès Y Takaoka TL Dayrat BA Boore JL Gosliner T Crawling through time: transition of snails to slugs dating back to the Paleozoic, based on mitochondrial phylogenomics Mar Genomics 2011 4 1 51 59 10.1016/j.margen.2010.12.006 21429465

[B4] Terrett JA Miles S Thomas RH Complete DNA sequence of the mitochondrial genome of *Cepaea nemoralis *(Gastropoda: Pulmonata) J Mol Evol 1996 42 2 160 168 10.1007/BF02198842 8919868

[B5] Hatzoglou E Rodakis GC Lecanidou R Complete sequence and gene organisation of the land snail *Albinaria coerulea* Genetics 1995 140 4 1353 1366 7498775 10.1093/genetics/140.4.1353 PMC1206699

[B6] Yamazaki N Ueshima R Terrett JA Yokobori S Kaifu M Segawa R Kobayashi T Numachi K Ueda T Nishikawa K Watanabe K Thomas RH Evolution of pulmonate gastropod mitochondrial genomes: comparisons of gene organizations of *Euhadra, Cepaea *and *Albinaria *and implications of unusual tRNA secondary structures Genetics 1997 145 3 749 758 9055084 10.1093/genetics/145.3.749 PMC1207859

[B7] Boore JL Animal mitochondrial genomes Nucleic Acids Res 1999 27 8 1767 1780 10.1093/nar/27.8.1767 10101183 PMC148383

[B8] Boore JL Medina M Rosenberg LA Complete sequences of the highly rearranged molluscan mitochondrial genomes of the scaphopod *Graptacme eborea *and the bivalve *Mytilus edulis* Mol Biol Evol 2004 21 8 1492 1503 10.1093/molbev/msh090 15014161

[B9] Grande C Templado J Cervera JL Zardoya R The complete mitochondrial genome of the nudibranch *Roboastra europaea *(Mollusca: Gastropoda) supports the monophyly of opisthobranchs Mol Biol Evol 2002 19 10 1672 1685 10.1093/oxfordjournals.molbev.a003990 12270894

[B10] Simison WB Boore JL Ponder WF, Lindberg DR Molluscan Evolutionary Genomics Phylogeny and evolution of the Mollusca 2008 Berkeley: University of California Press 447 461

[B11] Broughton RE Milam JE Roe BA The complete sequence of the Zebrafish (*Danio rerio*) mitochondrial genome and evolutionary patterns in vertebrate mitochondrial DNA Genome Res 2001 11 11 1958 1967 11691861 10.1101/gr.156801 PMC311132

[B12] Miya M Takeshima H Endo H Ishiguro NB Inoue JG Mukai T Satoh TP Yamaguchi M Kawaguchi A Mabuchi K Shirai SM Nishida M Major patterns of higher teleostean phylogenies: a new perspective based on 100 complete mitochondrial DNA sequences Mol Phylogenet Evol 2003 26 1 121 138 10.1016/S1055-7903(02)00332-9 12470944

[B13] Zhang P Chen YQ Zhou H Wang XL Qu LH The complete mitochondrial genome of a relic salamander, *Ranodon sibiricus *(Amphibia: Caudata) and implications for amphibian phylogeny Mol Phylogenet Evol 2003 28 3 620 626 10.1016/S1055-7903(03)00059-9 12927145

[B14] Morin PA Archer FI Foote AD Vilstrup J Allen EE Wade P Durban J Parsons K Pitman R Li L Bouffard P Abel Nielsen SC Rasmussen M Willerslev E Gilbert MT Harkins T Complete mitochondrial genome phylogeographic analysis of killer whales (*Orcinus orca*) indicates multiple species Genome Res 2010 20 7 908 916 10.1101/gr.102954.109 20413674 PMC2892092

[B15] Ki JS Hwang DS Park TJ Han SH Lee JS A comparative analysis of the complete mitochondrial genome of the Eurasian otter *Lutra lutra *(Carnivora; Mustelidae) Mol Biol Rep 2010 37 4 1943 1955 10.1007/s11033-009-9641-0 19757186

[B16] Jex AR Hu M Littlewood DT Waeschenbach A Gasser RB Using 454 technology for long-PCR based sequencing of the complete mitochondrial genome from single *Haemonchus contortus *(Nematoda) BMC Genomics 2008 9 11 10.1186/1471-2164-9-11 18190685 PMC2254599

[B17] Knapp M Hofreiter M Next generation sequencing of ancient DNA: requirements, strategies and perspectives Genes 2010 1 227 243 10.3390/genes1020227 PMC3954087 24710043

[B18] Meyer M Stenzel U Myles S Prüfer K Hofreiter M Targeted high-throughput sequencing of tagged nucleic acid samples Nucleic Acids Res 2007 35 15 e97 10.1093/nar/gkm566 17670798 PMC1976447

[B19] Dietz L Mayer C Arango CP Leese F The mitochondrial genome of *Colossendeis megalonyx *supports a basal position of Colossendeidae within the Pycnogonida Mol Phylogenet Evol 2010 58 3 553 558 21195785 10.1016/j.ympev.2010.12.016

[B20] Feldmeyer B Hoffmeier K Pfenninger M The complete mitochondrial genome of *Radix balthica *(Pulmonata, Basommatophora), obtained by low coverage shot gun next generation sequencing Mol Phylogenet Evol 2010 57 3 1329 1333 10.1016/j.ympev.2010.09.012 20875865

[B21] Miller W Drautz DI Janecka JE Lesk AM Ratan A Tomsho LP Packard M Zhang Y McClellan LR Qi J Zhao F Gilbert MT Dalén L Arsuaga JL Ericson PG Huson DH Helgen KM Murphy WJ Götherström A Schuster SC The mitochondrial genome sequence of the Tasmanian tiger (*Thylacinus cynocephalus*) Genome Res 2009 19 2 213 220 19139089 10.1101/gr.082628.108 PMC2652203

[B22] Cui Z Liu Y Li CP You F Chu KH The complete mitochondrial genome of the large yellow croaker, *Larimichthys crocea *(Perciformes, Sciaenidae): unusual features of its control region and the phylogenetic position of the Sciaenidae Gene 2009 432 1-2 33 43 10.1016/j.gene.2008.11.024 19100818

[B23] Duda M Kruckenhauser L Haring E Sattmann H Habitat requirements of the pulmonate land snails *Trochulus oreinos oreinos *and *Cylindrus obtusus *endemic to the Northern Calcareous Alps, Austria Eco mont 2010 2 2 10.1553/eco.mont-2-2s5 PMC4340512 25729612

[B24] Krajick K Nunataks, icebound islands of life Natl Geogr 1998 194 60 71

[B25] Frank C Frank C, Grillitsch H 2006 Plio-pleistozäne und holozäne Mollusken Österreichs Mitteilungen der prähistorischen Kommission 62 1998 Wien, Verlag der Österreichischen Akademie der Wissenschaften 397 861

[B26] Gittenberger E Piel WH Groenenberg DSJ The Pleistocene glaciations and the evolutionary history of the polytypic snail species *Arianta arbustorum *(Gastropoda, Pulmonata, Helicidae) Mol Phylogenet Evol 2004 30 1 64 73 10.1016/S1055-7903(03)00182-9 15022758

[B27] Arthofer W Cadahía L Kruckenhauser L Ten new microsatellite loci for analysis of genetic diversity in isolated populations of the Alpine land snail *Cylindrus obtusus* Conserv Genet 2009 11 3 1115 1118

[B28] Folmer O Black M Hoeh W Lutz R Vrijenhoek R DNA primers for amplification of mitochondrial cytochrome c oxidase subunit I from diverse metazoan invertebrates Mol Mar Biol Biotech 1994 3 5 294 299 7881515

[B29] Merritt TJ Shi L Chase MC Rex MA Etter RJ Quattro JM Universal cytochrome b primers facilitate intraspecific studies in molluscan taxa Mol Mar Biol Biotech 1998 7 1 7 11 9597773

[B30] Rozen S Skaletsky HJ Krawetz S, Misener S Primer3 on the WWW for general users and for biologist programmers Bioinformatics Methods and Protocols: Methods in Molecular Biology 2000 Totowa, NJ: Humana Press 365 386 10.1385/1-59259-192-2:365 10547847

[B31] Wyman SK Jansen RK Boore JL Automatic annotation of organellar genomes with DOGMA Bioinformatics 2004 20 17 3252 3255 10.1093/bioinformatics/bth352 15180927

[B32] Lowe TM Eddy SR tRNAscan-SE: a program for improved detection of transfer RNA genes in genomic sequence Nucleic Acids Res 1997 25 5 955 964 9023104 10.1093/nar/25.5.955 PMC146525

[B33] Laslett D Canbäck B 2008 ARWEN: a program to detect tRNA genes in metazoan mitochondrial nucleotide sequences Bioinformatics 2008 24 2 172 175 10.1093/bioinformatics/btm573 18033792

[B34] DeJong RJ Emery AM Adema CM The mitochondrial genome of *Biomphalaria glabrata *(Gastropoda: Basommatophora), intermediate host of *Schistosoma mansoni* J Parasitol 2004 90 5 991 997 10.1645/GE-284R 15562597

[B35] Glenn TC Field guide to next-generation DNA sequencers Mol Ecol Res 2011 11 759 769 10.1111/j.1755-0998.2011.03024.x 21592312

[B36] Willerslev E Gilbert MT Binladen J Ho SY Campos PF Ratan A Tomsho LP da Fonseca RR Sher A Kuznetsova TV Nowak-Kemp M Roth TL Miller W Schuster SC Analysis of complete mitochondrial genomes from extinct and extant rhinoceroses reveals lack of phylogenetic resolution BMC Evol Biol 2009 9 95 10.1186/1471-2148-9-95 19432984 PMC2694787

[B37] Simon SA Zhai J Nandety RS McCormick KP Zeng J Mejia D Meyers BC Short-read sequencing technologies for transcriptional analyses Annu Rev Plant Biol 2009 60 305 333 10.1146/annurev.arplant.043008.092032 19575585

[B38] Watanabe Y Tsurui H Ueda T Furushima R Takamiya S Kita K Nishikawa K Watanabe K Primary and higher order structures of nematode (*Ascaris suum*) mitochondrial tRNAs lacking either the T or D stem J Biol Chem 1994 269 36 22902 22906 8077242

[B39] Yokobori S Pääbo S Transfer RNA editting in land snail mitochondria Proc Natl Acad Sci USA 1995 92 22 10432 10435 10.1073/pnas.92.22.10432 7479799 PMC40811

[B40] Morrison DA How and where to look for tRNAs in Metazoan mitochondrial genomes, and what you might find when you get there Online Archive arXiv.org, ID:arXiv:1001:3813v1 [q-bio-GN], 2010

[B41] Rawling TA MacInnis MJ Bieler R Boore JL Collins TM Sessile snails, dynamic genomes: gene rearrangements within the mitochondrial genome of a family of caenogastropod molluscs BMC Genomics 2010 11 440 10.1186/1471-2164-11-440 20642828 PMC3091637

[B42] Wolstenholme DR Animal mitochondrial DNA: structure and evolution Int Rev Cytol 1992 141 173 216 1452431 10.1016/s0074-7696(08)62066-5

[B43] Masta SE Boore JL The complete mitochondrial genome sequence of the spider *Habronattus oregonensis *reveals rearranged and extremely truncated tRNAs Mol Biol Evol 2004 21 8 893 902 15014167 10.1093/molbev/msh096

[B44] Dellaporta SL Xu A Sagasser S Jakob W Moreno MA Buss LW Schierwater B Mitochondrial genome of *Trichoplax adhaerens *supports Placozoa as the basal lower metazoan phylum Proc Natl Acad Sci USA 2006 103 8751 8756 10.1073/pnas.0602076103 16731622 PMC1470968

[B45] Kurabayashi A Ueshima R Complete sequence of the mitochondrial DNA of the primitive opisthobranch *Pupa strigosa*: systematic implications of the genome organization Mol Biol Evol 2000 17 2 266 277 10.1093/oxfordjournals.molbev.a026306 10677849

[B46] Negrisolo E Babbucci M Patarnello T The mitochondrial genome of the ascalaphid owlfly *Libelloides macaronius *and comparative evolutionary mitochondriomics of neuropterid insects BMC Genomics 2011 12 221 10.1186/1471-2164-12-221 21569260 PMC3115881

[B47] Harris DJ Can you bank on GenBank? Trends Ecol Evol 2003 18 7 317 319 10.1016/S0169-5347(03)00150-2

[B48] Groenenberg DSJ Neubert E Gittenberger E Reappraisal of the "Molecular phylogeny of Western Palaearctic Helicidae *s.l*. (Gastropoda: Stylommatophora)": when poor science meets GenBank Mol Phylogenet Evol 2011 61 914 923 10.1016/j.ympev.2011.08.024 21930220

